# Designing a School-Based, Complex Public Health Intervention to Improve Iodine Awareness in Adolescents in Six Countries

**DOI:** 10.3390/nu18172820

**Published:** 2026-08-28

**Authors:** Bodil Just Christensen, Natalia Cecon-Stabel, Synnøve Næss Sleire, Lisbeth Dahl, Signe Svarrer Skovgaard-Pedersen, Vivien Henck, Phil Pendt, Muhammad Nasir Khan Khattak, Elias Peschke, Henry Völzke, Mithila Faruque, Rehman Mehmood Khattak, Aisha Imtiaz, Muhammad Altaf Khan, Georgia Soursou, Konstantinos C. Makris, Simona Gaberšček, Katja Zaletel, Jayne V. Woodside, Sarah C. Bath, Linda Henderson, Anna Bokor, Joyce Greene, Deqa Jama, Freia De Bock, Gitte Ravn-Haren

**Affiliations:** 1 National Food Institute, Technical University of Denmark, DK-2800 Kongens Lyngby, Denmark; sigha@food.dtu.dk (S.S.S.-P.); girh@food.dtu.dk (G.R.-H.); 2 Department of Biomedical Sciences, University of Copenhagen, DK-2200 Copenhagen, Denmark; 3 Department of General Pediatrics, Neonatology and Pediatric Cardiology, Medical Faculty, University Hospital Düsseldorf, Heinrich-Heine-University Düsseldorf, DE-40225 Düsseldorf, Germany; n.cecon@hotmail.de (N.C.-S.); freia.debock@med.uni-duesseldorf.de (F.D.B.); 4 Seafood, Nutrition and Environmental State, Institute of Marine Research (IMR), NO-5005 Bergen, Norway; synnoeve.naess@hi.no (S.N.S.); lisbeth.dahl@hi.no (L.D.); 5 Institute for Community Medicine, University Medicine Greifswald, DE-17475 Greifswald, Germany; vivien.henck@uni-greifswald.de (V.H.); phil.pendt@uni-greifswald.de (P.P.); muhammadnasir.khan@med.uni-greifswald.de (M.N.K.K.); elias.peschke@uni-greifswald.de (E.P.); voelzke@uni-greifswald.de (H.V.); 6 Department of Noncommunicable Diseases, Faculty of Public Health, Bangladesh University of Health Sciences, Dhaka Dh-1207, Bangladesh; mithilafaruque@buhs.ac.bd; 7 Department of Zoology, Islamia College Peshawar, Peshawar PK-25000, Pakistan; rehman.mehmood@icp.edu.pk (R.M.K.); aishaimtiaz@icp.edu.pk (A.I.); muhammadaltafkhan@icp.edu.pk (M.A.K.); 8 CLOTHO Precision Exposomics and Health Laboratory, Cyprus International Institute for Environmental and Public Health, School of Health Sciences, Cyprus University of Technology, CY-3041 Limassol, Cyprus; georgia.soursou@cut.ac.cy (G.S.); konstantinos.makris@cut.ac.cy (K.C.M.); 9 Department of Rehabilitation Sciences, School of Health Sciences, Cyprus University of Technology, CY-3041 Limassol, Cyprus; 10 Division of Nuclear Medicine, University Medical Centre Ljubljana, SI-1525 Ljubljana, Slovenia; simona.gaberscek@kclj.si (S.G.); katja.zaletel@kclj.si (K.Z.); 11 Faculty of Medicine, University of Ljubljana, SI-1000 Ljubljana, Slovenia; 12 Centre for Public Health, Queen’s University Belfast, Belfast BT12 6BA, UK; j.woodside@qub.ac.uk; 13 Faculty of Health and Medical Sciences, University of Surrey, Guildford GU2 7XH, UK; s.bath@surrey.ac.uk; 14 Thyroid Federation International, Transpolispark, Siriusdreef 17-27, NL-2132 WT Hoofddorp, The Netherlands; linda.henderson@thyroid-federation.org (L.H.); anna.bokor@thyroid-federation.org (A.B.); 15 Iodine Global Network, P.O. Box 51030, 375 des Epinettes, Ottawa, ON K1E 3E6, Canada; jgreene@ign.org (J.G.); djama@ign.org (D.J.)

**Keywords:** food literacy, food competencies, educational intervention, active learning, ABC of iodine educational program, iodine nutrition, iodine deficiency

## Abstract

**Background**: Iodine is an essential micronutrient required for foetal development, cognitive function, and metabolic regulation; however, suboptimal iodine status remains a public health concern in Europe and other regions. Improving food literacy related to iodine may support healthier dietary choices during adolescence, a critical life stage for establishing long-term habits. This intervention development study describes the development of *The ABC of Iodine* Teaching Programme within the EUthyroid2 project, designed to enhance iodine-related knowledge and awareness among adolescents aged 13–17 years across six regions (UK, Republic of Cyprus, Slovenia, Germany, Bangladesh, and Pakistan). **Methods**: The intervention was developed according to the Behaviour Change Wheel, targeting capability, opportunity, and motivation, and informed by guidance for complex interventions. The programme was comprised of three flexible, culturally adapted modules integrating lectures on iodine physiology, deficiency risks, WHO recommendations, and locally relevant dietary sources. Active learning strategies, including collaborative tasks and personalised feedback through an Iodine Feedback Tool, were included. Materials were translated and adapted to local contexts and implemented in a hybrid format combining printed booklets with QR-linked digital resources. **Results**: The primary outcome of the intervention development process was *The ABC of Iodine* Teaching Programme, a multi-component, scalable educational intervention aligned with principles of food literacy, active learning and behaviour change theory. It incorporated behaviour change techniques and context-specific adaptations to facilitate engagement and knowledge acquisition in school settings. The programme is currently under evaluation in participating regions. **Conclusions**: This study presents the systematic development of a complex teaching programme. By combining behaviour change theory with innovative and context-sensitive educational strategies, *The ABC of Iodine* Teaching Programme provides a flexible and scalable framework for improving iodine-related food literacy among adolescents. Its effectiveness will be determined through the ongoing evaluation studies.

## 1. Introduction

Enhancing public understanding of iodine nutrition is a central strategy in the prevention of iodine deficiency disorders (IDD), which continue to pose a significant global health challenge, particularly among women of reproductive age. As a key component in the synthesis of thyroid hormones, iodine ensures proper neurological development during pregnancy and childhood, regulates metabolism, and supports physical growth throughout life [1]. Although many countries have implemented iodine fortification policies, such as universal salt iodisation, studies indicate that mild to moderate iodine deficiency persists across Europe [2,3] and beyond [4,5].

Adolescents represent a particularly important group for nutritional education, as habits and knowledge formed during this age can influence long-term health outcomes [6]. Considering this, the EUthyroid2 consortium developed a targeted educational intervention designed to raise awareness and knowledge of the dietary importance of iodine among adolescents [7]. The aim of this intervention development study is to present the conceptual framework, development, and contextual adaptation of the teaching resources created for intervention implementation.

### 1.1. Improved Nutrition Through Increased Awareness

Behaviour change interventions often aim to encourage healthier behaviours by enhancing knowledge and understanding of specific health-related topics. Studies show that raising awareness through education about the importance of nutrients and their dietary sources can lead to improved dietary choices [8,9]. For example, school-based interventions that educate children about nutrition and micronutrients have demonstrated increased nutritional knowledge and diet quality outcomes [10,11]. Moreover, including information about nutrient sources and locally available foods can enhance the effectiveness of these interventions [12].

School settings have been consistently identified as promising environments for nutrition promotion, as they provide access to large adolescent populations and allow health education to be integrated into existing educational structures [13]. Recent reviews indicate that school-based nutrition interventions are most effective when they are embedded within the curriculum, combine multiple intervention components, and incorporate interactive and behaviour-orientated learning approaches rather than relying solely on the transfer of factual knowledge [12,13,14,15].

Educational interventions that incorporate behaviour change techniques such as feedback, social support, and self-monitoring tend to have stronger and longer-lasting effects on behaviour than those solely focused on knowledge dissemination [16]. Multi-component interventions have proved to be more effective when combining awareness-raising with skill-building and environmental support [12].

These findings align with recent developments within the field of food literacy, which conceptualises nutrition-related competence as more than factual knowledge of nutrients and foods. Food literacy encompasses the functional, relational and critical capacities required to interpret nutrition information, navigate food environments and translate knowledge into everyday dietary practices. Consequently, adolescent nutrition interventions increasingly emphasise participatory and experiential learning approaches that combine knowledge acquisition with reflection, skill development and social engagement [17,18].

Despite growing recognition of adolescent nutrition as a public health priority, important knowledge gaps remain [12]. Much of the existing intervention literature has focused on obesity prevention, general healthy eating behaviours, or fruit and vegetable consumption [13], whereas comparatively little attention has been directed towards micronutrient-specific educational interventions. Furthermore, few studies have examined how nutrition education programmes can be systematically adapted and implemented across diverse cultural and educational contexts while maintaining a common theoretical foundation and evaluation framework [14].

The present intervention was developed to address these gaps by combining behaviour change theory, food literacy principles, active learning approaches, and systematic contextual adaptation within a multinational implementation framework focused specifically on iodine awareness.

### 1.2. The EUthyroid2 Consortium: Projects and Intervention Design

EUthyroid2 is an ongoing Horizon Europe-funded consortium that builds on previous research to strengthen public health responses to iodine-related challenges [19]. The consortium projects focus on adolescents (13–17 years old) and young women (18–25 years old). This article reports exclusively on the intervention conducted among adolescents in an educational setting; details on the parallel intervention among young women can be found in the protocol publication (N. Cecon-Stabel, S. Sleire, L. Dahl et al., in preparation). The aims of the study were to test educational strategies for promoting long-term iodine awareness and improved iodine status and to explore scalable models for delivering population-level iodine education. The interventions were implemented in educational settings in six regions, including the United Kingdom, the Republic of Cyprus, Slovenia, Germany, Bangladesh, and Pakistan, allowing for cross-cultural insights into best practices (see [7] and [19] for more details on the EUthyroid2 projects).

The intervention was carried out in secondary schools, high schools, and vocational schools and consisted of a flexible three-module teaching programme, *The ABC of Iodine*. Modules could be delivered individually or in combination, allowing intervention duration to range from a few teaching hours to several weeks depending on module combination. In Module A, the teaching programme combined a lecture on iodine’s physiological role, health consequences of iodine deficiency, recommended dietary intake, and main dietary sources with participatory learning and culturally relevant iodine-rich food examples to strengthen both awareness and practical understanding. Students’ awareness and knowledge of iodine were assessed at baseline, followed by two follow-up assessments conducted two to four weeks after completion of the intervention and again after six to eight months. The in-class activities were supported by an online learning platform where teachers and students could access all information and materials (for a more detailed description see [7]). Before any teaching began, all students were introduced to the Iodine Feedback Tool, which served as a motivational activity, prompting curiosity in iodine intake. This was a brief questionnaire consisting of four questions, asking about the students’ consumption of key iodine sources (iodised salt, dairy, fish, and supplements). The tool provided students with automated, personalised feedback on their current intake patterns, along with practical recommendations for portion sizes of iodine-rich foods. The teaching programme was considered a complex intervention because it combined multiple interacting components: knowledge-building, self-assessment and feedback, participatory activities, and contextual adaptations. Evidence shows that such multi-component approaches are generally more effective than single-focus strategies, as they address both individual, social, and contextual parameters, leading to stronger and more sustainable outcomes [8,9]. The intervention was developed with careful consideration of and adaptation to socio-cultural factors to ensure relevance across the different geographic localities encompassed in the project [13]. Additionally, Modules B and C implemented an active learning approach, emphasising engagement through collaborative activities, allowing the effectiveness of active learning approaches to be assessed and compared with traditional lecture-based instruction in Module A as part of the subsequent effect evaluation [20].

## 2. Materials and Methods

### 2.1. Theoretical Background for Intervention Development

This methodological study describes the theory-informed development, contextual adaptation, and planned evaluation of a complex teaching programme to improve iodine awareness among adolescents.

**Figure 1:**
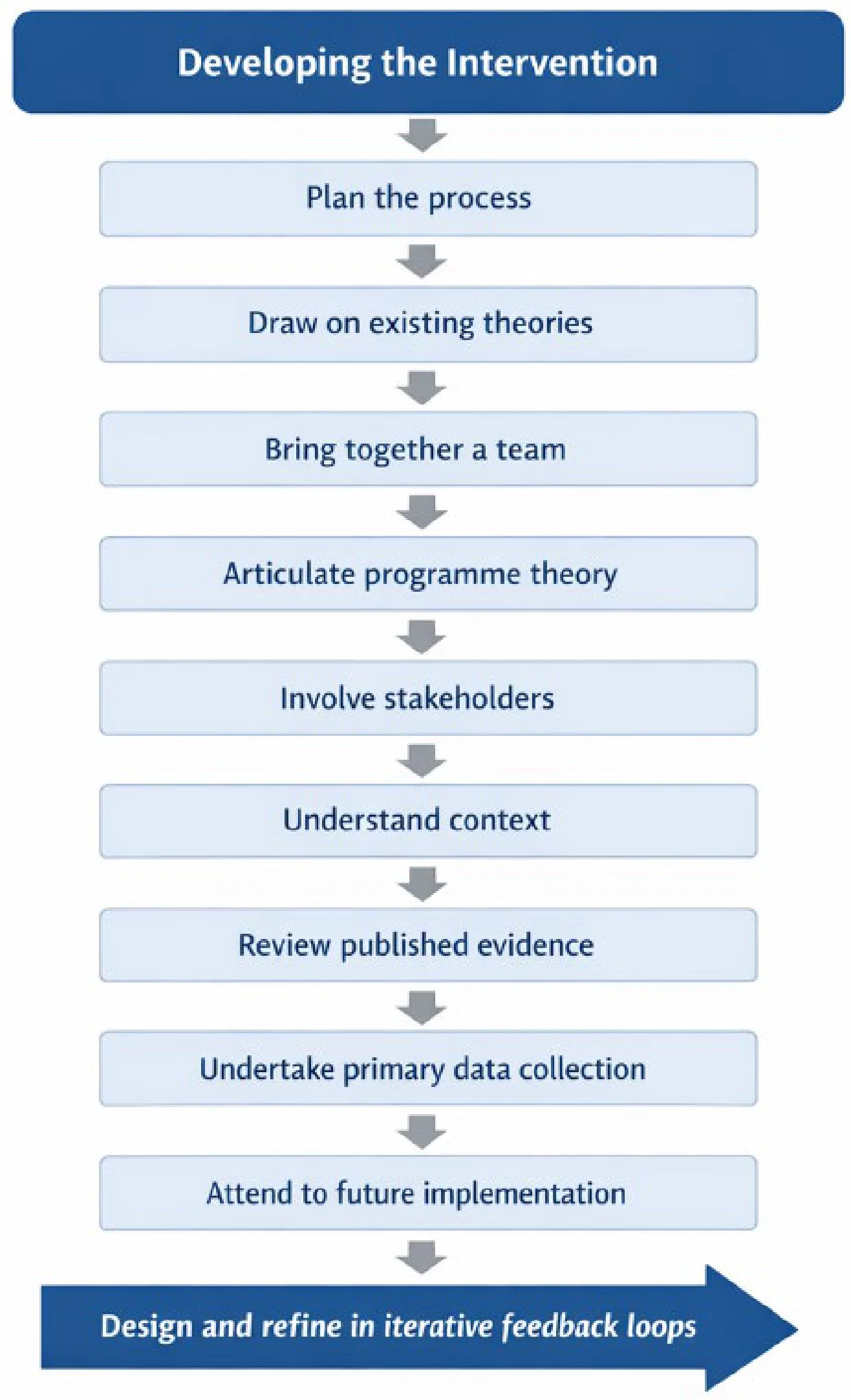
Intervention development process for The ABC of Iodine Teaching Programme, adapted from O’Cathain et al.’s framework for developing complex health interventions.

The development of the intervention was guided by O’Cathain et al.’s (2021) approach on how to develop complex interventions that improve health and health care outcomes [21]. The authors emphasised that developing complex health interventions requires making the underlying programme theory explicit. This involves clearly articulating how and why the intervention is expected to work, identifying its components, mechanisms, and contextual factors, and mapping these in a coherent model. The development should preferably unfold as an iterative, evidence-informed process that integrates stakeholder perspectives and feasibility testing, ensuring that the intervention is coherent and adaptable to the specific intervention context(s). Further, the evaluation strategy and objectives should be defined early to secure alignment between design and assessment [21]. In developing *The ABC of Iodine* teaching programme, these principles structured the design, though some steps were reordered due to practical concerns (see Figure 1). All elements were retained, and the recommendation to establish evaluation aims from the outset was particularly influential, given that assessing the most effective teaching modalities was a primary goal of the project.

To operationalise the intervention objective, the following primary research question was formulated:

Does the teaching programme on iodine in the selected educational settings increase iodine awareness in students aged 13–17 from baseline to follow-up (2–4 weeks post-intervention and at 6–8 months)?

This question can be further unfolded into the following sub-questions:

- Are some teaching modalities more effective than others in increasing iodine awareness?
- Do specific teaching modalities affect the retention of increased iodine awareness among students?
- Does the educational setting (comparing vocational schools, high schools, and secondary schools) influence the potential increase in iodine awareness among students?
- Are there differences in outcomes regarding iodine awareness among students when considering various age groups and gender distinctions?
- Does increased iodine awareness lead to behaviour change and healthier iodine intake habits?

Development of the intervention components addressed various simultaneous aims: (1) To test if iodine awareness could be improved through the teaching programme, to what degree and with which retention rate, and (2) to provide evidence for which elements and teaching modalities worked and how effectively. A first step in achieving these objectives was to draw on the Behaviour Change Wheel (BCW) and its underlying COM-B model [22], where behaviour (the “B”) is understood as the result of three interacting components: Capability, Opportunity, and Motivation. The COM-B framework was used to identify relevant intervention functions and propose corresponding elements aimed at creating the conditions necessary for behaviour change. The design additionally considered the strengthening of teachers’ awareness and behavioural capability to subsequently improve the students’ awareness and behavioural capability.

### 2.2. Practical Organisation and the Design Team

The organisation of the intervention development was shaped by the overall project protocol, which specified that the intervention and its constituent elements should be conceptualised by an international and interdisciplinary work package (WP2) team [19]. The design team consisted of six researchers based in Norway, Germany, and Denmark, combining a broad range of expertise. This included specialised knowledge of iodine and nutrition, clinical medicine, social science, intervention research, development of educational material, and both practical experience and theoretical grounding in psychological aspects of behaviour change. This intervention development team proved highly functional, and the various expertise complemented each other in a beneficial manner. The team was tasked with designing and coordinating the two intervention arms to be implemented in healthcare and educational settings, respectively. They first met for a workshop in early 2023, shortly after the project initiation. At this workshop, the team was consolidated, the initial steps in the intervention design were agreed upon, and a biweekly online meeting structure was established. These regular meetings formed the backbone of the coordination and refinement of the two interventions, allowing the team to continuously align the design, development processes, and the concrete intervention components.

In March 2023, the full project consortium (comprising partners and participants from the eight study regions covering both healthcare and educational settings) met for a kick-off meeting. This meeting provided an opportunity to present, discuss, and agree on initial approaches, conceptual models, and preliminary design considerations across work packages. It also allowed for the identification of relevant gatekeepers and key partners in each local context. On this organisational basis, the WP2 design team then began developing the specific intervention components, ensuring that these were aligned with the BCW framework and selected intervention functions.

### 2.3. Intervention Functions and Tools

The Iodine Feedback Tool was designed as a motivational trigger, increasing students’ awareness of their own iodine intake and sparking curiosity about both the recommended intake level and concrete dietary practices required to achieve a sufficient iodine intake. The individualised feedback, and thereby the higher level of personalisation [9], has been shown to increase motivation, therefore increasing effectiveness [23].

The Iodine Feedback Tool consisted of four questions assessing the average intake of the four most important dietary sources of iodine globally: iodised salt, iodine supplements, milk and dairy products, and fish (see Supplementary Material S1: Iodine Feedback Tool). The tool was developed from a validated, digital, iodine-specific dietary screener [24]. Each question offered two or more response options regarding frequency of intake, with each option assigned a specific score. Since countries in the study regions differed in both iodine sources and iodine content of those sources, the scoring for certain questions in the Iodine Feedback Tool was adapted to each country. The scoring for milk and dairy products, fish, and iodine supplements was identical across countries, whereas the iodised salt score was adapted according to national fortification practices and iodine concentrations in salt. Specifically, the use of iodised salt contributed positively to the “healthy iodine habits” score in Bangladesh, Pakistan, and Slovenia, where iodised salt makes a substantial contribution to population iodine intake, whereas it was assigned a score of 0 in the UK and Cyprus due to the low and variable iodine content of salt in these regions. The overall score was then used to guide interpretation and provide feedback to participants. Scores of 0–1 point indicated that more iodine-rich foods should be included in the diet, whereas a score of 2 or more points suggested that sufficient dietary sources of iodine were already being consumed. It should be noted that the Iodine Feedback Tool was not intended to estimate iodine intake but rather to provide brief motivational feedback on the dietary sources of iodine included in the participant’s diet. Although the scoring system cannot fully account for all regional variation in the iodine content of foods, this limitation reflects the lack of comparable country-specific food composition data rather than methodological shortcomings. Given the available evidence, the tool provides a pragmatic and context-sensitive assessment of iodine-related dietary behaviours.

The subsequent lecture, individual exercises, and group assignments were organised as complementary modalities of learning that targeted enablement and education [22] through testing (quizzes, short assignments) and inquiry-based (“learning-by-doing”) activities, intended to deepen both declarative and procedural forms of knowledge [25] and to stage peer-based modelling in the classroom [22].

Establishing intended learning outcomes (ILOs) was the next step to ensure alignment between teaching modalities and evaluation, creating a coherent thread throughout the intervention design. By first specifying what students should learn (e.g., describing iodine’s bodily functions, its health significance particularly in pregnancy, intake recommendations, deficiency consequences, and dietary applications), the evaluation could then test which modalities best facilitated these objectives.

**Figure 2:**
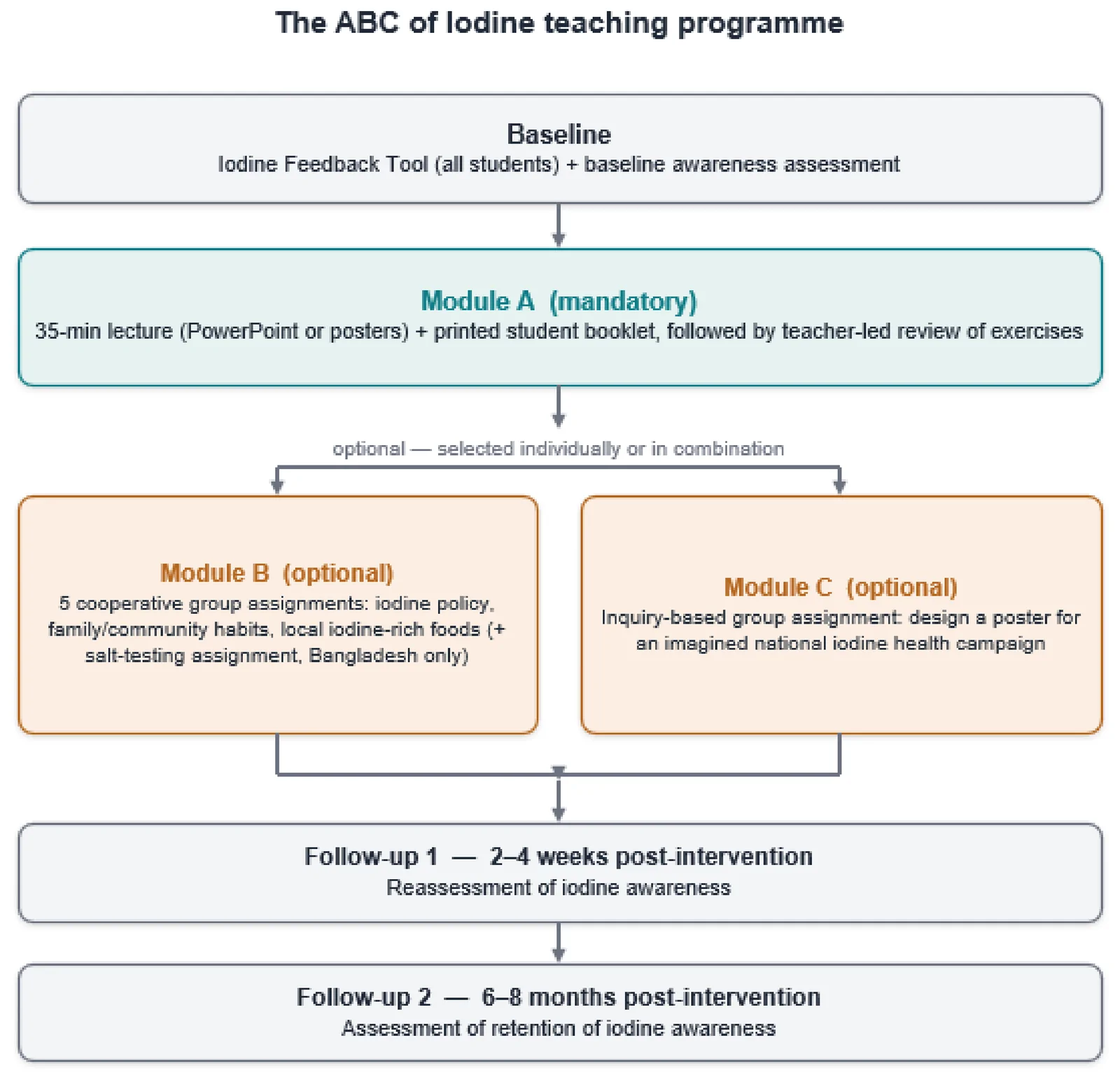
Scheme of *The ABC of Iodine* teaching programme, showing intervention timing, mandatory and optional modules, and assessment timepoints.

To enable the required differentiated evaluation of the various elements and pedagogical modalities’ effectiveness, a flexible three-step model with three modules (A, B and C) was developed, allowing teachers to select and combine components as needed (Figure 2). This model allowed researchers to compare which learning outcomes and retention rates were associated with different stand-alone or combinations of teaching elements. Thus, implementation would intentionally vary across schools; the study therefore compared different implementation trajectories and examined both process and feasibility. The analytic focus was on identifying which trajectories appeared most promising for fostering sustained iodine awareness amongst students.

**Figure 3:**
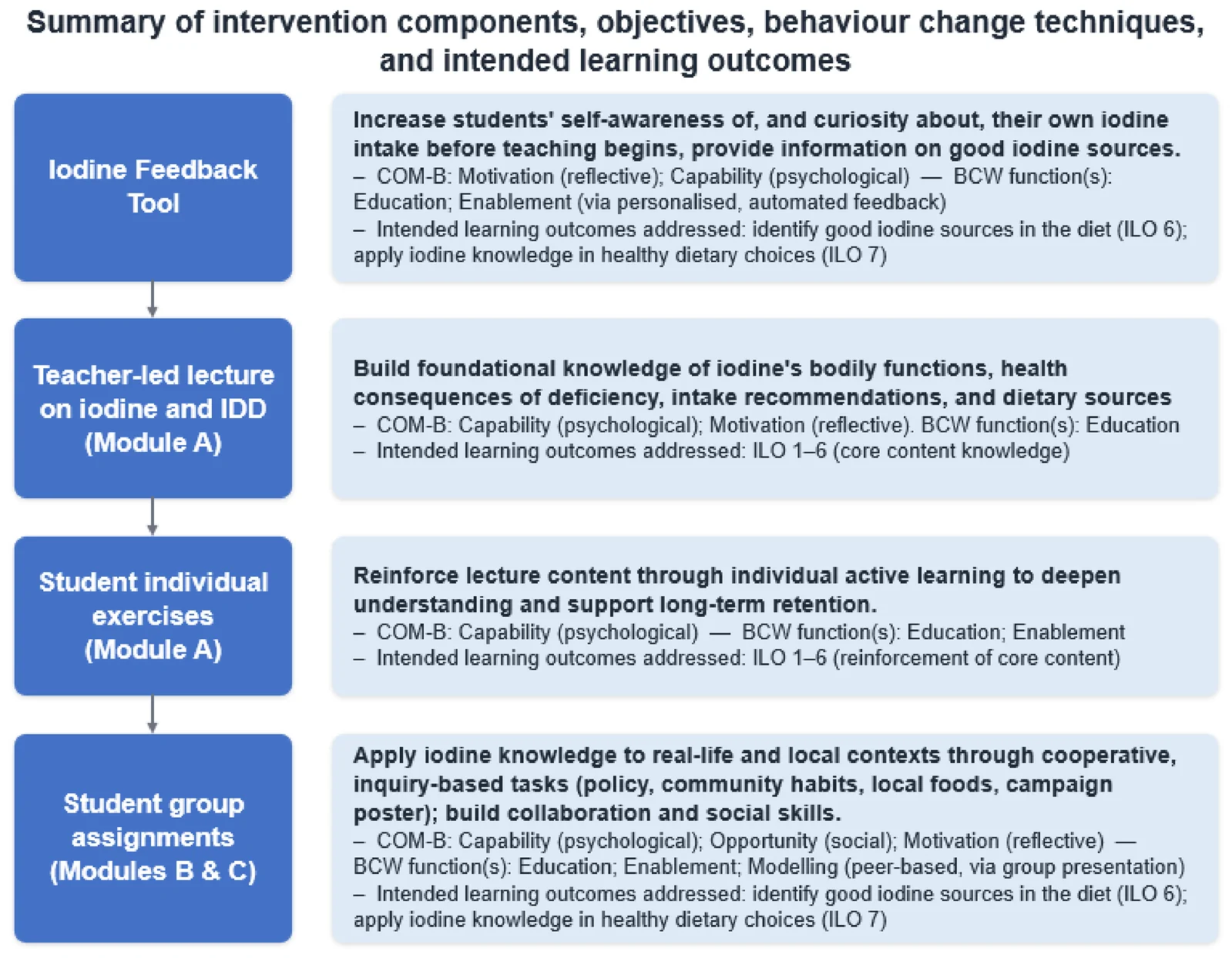
Overview of the ABC of Iodine intervention components, their objectives, and the corresponding COM-B target(s) and Behaviour Change Wheel (BCW) intervention function(s) they address, together with the intended learning outcomes (Table 1) each component is designed to support. COM-B = Capability, Opportunity, Motivation-Behaviour model; BCW = Behaviour Change Wheel.

**Module A** was mandatory and consisted of a 35-min classroom lecture, delivered either via PowerPoint or with posters that summarised key education points on iodine and health. The teaching was supported by a printed student booklet that presented the lecture content in written form and listed the subsequent exercises (teaching materials are available at https://euthyroid2.eu/abc-of-iodine/language/, accessed on 1 August 2026). After the lecture, a teacher-led review session of the exercises, which was designed to reinforce the material through active learning, took place. **Module B** was optional and offered five cooperative learning assignments. The assignments covered topics such as iodine policies, iodine knowledge, health habits in the family or community, and local availability of iodine-rich foods. In Bangladesh, an additional sixth assignment was included, in which students brought salt from home and tested its iodine content. This hands-on activity helped students connect classroom learning to their own daily lives and understand why iodine fortification matters. Students worked in groups to complete their assigned tasks and subsequently presented their work to the class. This structure allowed different groups to address different topics while ensuring that all students were exposed to the full range of the teaching programme. **Module C** was also optional and introduced an inquiry-based assignment in which student groups designed a poster for an imagined national public health campaign on iodine guided by examples drawn from real campaigns. This assignment encouraged creativity and critical thinking and was designed to deepen students’ conceptual understanding of the topic (for a graphic overview see Figure 3).

The module design was selected not only to address the intervention’s research questions but also to provide teachers flexibility in integrating the intervention into existing curriculum and the entailed constraints.

### 2.4. Adjustment to Regional Contexts

To adjust this preliminary intervention design to actual teaching settings and specific socio-cultural contexts in the six intervention regions, a two-phase context assessment was carried out using the CICI framework [26]. To inform this process, the intervention region leads and identified researchers at the kick-off meeting were involved, as they possessed context-specific knowledge about dietary habits, youth culture and norms, and features of local educational settings, including institutional structures and the language of instruction. In the first phase, stakeholders completed a questionnaire based on CICI dimensions (context, implementation and setting); their responses then formed the basis for online workshops with each study region, where identified questions and challenges were discussed and possible solutions jointly developed. These discussions were summarised in written form and returned to the regions for comment, thus establishing a partial co-design process [27]. Stakeholder feedback was systematically reviewed and was instrumental in shaping the flexible intervention design. In particular, stakeholders highlighted substantial variation in the time available for nutrition education across regions, which led to the development of the three-module structure that allowed implementation to be adapted to local educational contexts while maintaining common learning objectives. Several country-specific elements (e.g., national differences in iodine policies or variation in dietary patterns and thus in common iodine-rich foods) were mapped. Further, the assessment revealed marked regional differences in school systems, teaching practices, curriculum structures, and broader educational frameworks, which underscored the need for the flexible intervention model. In response, the three modules were subsequently adjusted to accommodate variation in institutional structures, available time resources, and preferred teaching modalities. Although implementation was adapted to local educational contexts, the core intervention content remained identical across study regions. All educational materials were based on the same learning objectives and key messages, while implementation consistency was further supported through detailed teacher guidelines and standardised instructions for delivery.

As an example, the lecture and student exercises in mandatory Module A were shortened to better fit curriculum structures, notably in the UK.

The design of the platform was inspired by interactive environments developed in EU-funded projects and nutrition-focused e-learning tools, including recent work on gamified nutrition education [28]. Development of the case-based assignments in Modules B and C was informed by theories of active and cooperative learning, emphasising students’ involvement through hands-on tasks, critical reflection, and collaboration, which are associated with improved understanding and retention [29]. Case-based and group assignments (see booklet p 20ff, assignments B1–B4 and C at https://euthyroid2.eu/abc-of-iodine/abc/students/en/, accessed on 1 August 2026) therefore required students to research, analyse, and discuss iodine-related issues and to present their work to peers. These active learning techniques (e.g., case studies and collaborative problem-solving) shifted the focus from the more passive information transmission in Module A to active knowledge construction in both Modules B and C. This structure was adopted to evaluate research sub-questions (a) and (b), which addressed the modular teaching structure, and to test if, for example, Module A content alone would be ‘sufficient’ for sustained awareness, or if collaborative problem-solving, as exemplified by Modules B and C, would be needed for deeper learning and improved retention span.

### 2.5. Didactical Design: Online Learning Platform and Case-Based Assignments

*The ABC of Iodine* platform, an online learning forum in the intervention, was chosen as a primary access point for all teacher and student information and materials. The platform supported active and self-directed learning, enabling students to construct knowledge through interaction and problem-solving [30]. The multimodal design, which combined text with a variety of tasks, accommodated different learning styles and supported both blended and hybrid learning formats, making the materials adaptable to diverse local contexts. In practical terms, the platform enabled scalability across geographic and linguistic boundaries, which was vital for EUthyroid2’s cross-national implementation [31]. Further, a secondary aim was to ensure standardisation of content and learning objectives while facilitating data collection for monitoring and evaluation.

### 2.6. Pre-Test Feedback, Adaptions, and Improvements

Selected teaching materials (a draft of the student booklet, an outline of the structure and content of the online platform, and suggested PowerPoint slides for the lecture) were pretested and evaluated by two experienced teachers from the UK, who also received a general introduction to the intervention design and its theoretical foundations. Through online semi-structured evaluation interviews, they provided detailed feedback on the overall concept and structure as well as on the draft materials; the interviews were recorded, transcribed, and summarised, and all substantive points were used to revise the intervention modalities and teaching resources. Key recommendations included printing the booklet and revising the graphic design by reducing text and increasing visual elements. It was also suggested to use motivational triggers at the beginning of the lesson. Teachers recommended providing brief activities for students who finished earlier (extension tasks), such as word searches that revisited or introduced key concepts. Additionally, they proposed producing posters with concise summaries that could be used in classrooms where PowerPoint was not feasible, as well as serving as visual reminders.

Based on the pre-test, it was decided that the booklet should be printed and handed out to students in enrolled schools, as a physical booklet was expected to foster a stronger sense of ownership and support deeper cognitive engagement with the material [32,33]. In addition, the printed booklet could serve as a fixed anchor for QR codes and links to the digital platform, which students could access on their own devices. Extension tasks for strong students (short drawing and writing exercises designed for use during waiting time) were also readily available.

The decision to provide each student with a printed version of the booklet had several practical consequences and prompted further adaptations. The original online booklet was relatively long and would have been costly to print. It was also considered wasteful to produce multiple pages of information, examples, and supplementary resources for Modules B and C when only Module A was mandatory. As a result, economic constraints led to a hybrid design in which the printed booklet contained the core compulsory components from Module A along with a brief introduction to Modules B and C. More detailed explanations, guidelines, and scaffolding resources for the optional modules were made available online via QR codes, allowing teachers who wished to use the additional modules to access them easily. This approach also enabled teachers to print the assignments in settings where students did not have access to the internet.

In parallel, a set of educational posters was developed, and the teaching videos originally envisaged as preparatory training for teachers were abandoned due to time and translation constraints and replaced with an extended written version of the teacher guidelines. A further practical challenge concerned the iodine test kits for one of the Module B assignments. These kits, supplied by UNICEF, could only be readily delivered to Bangladesh, meaning that the salt-testing assignment was feasible there but not in the European study regions. For Pakistan, delivery would, in principle, have been possible, but the procurement and distribution procedures proved so complex that the assignment was ultimately omitted. The UK offered gift vouchers for the best posters created in Module C, while Pakistan implemented both poster and survey competitions with tiered incentives. Participating teachers and students will also be invited for a formal celebration ceremony. In Bangladesh, both the survey and poster competitions were not possible to implement due to institutional infrastructure, class schedules and lack of teacher involvement.

### 2.7. Development of Process Evaluation Questionnaire (PEQ)

To be able to evaluate the project’s hypothesis that certain teaching modalities may be more effective than others in fostering sustained iodine awareness, a Process Evaluation Questionnaire (PEQ) was developed separately for students and teaching staff (see Supplementary Material S2: Process Evaluation Questionnaire for students and Supplementary Material S3: Process Evaluation Questionnaire for teachers and teaching staff). The objective of the questionnaires was to evaluate implementation feasibility by identifying contextual factors potentially hindering intervention delivery, such as student motivation, teacher preparedness, material access, or technical issues like unstable internet connectivity. The PEQ was developed to complement the Awareness Questionnaire by identifying potential explanations for variation in intervention effectiveness and implementation across settings.

Informed by the RE-AIM framework’s dimensions (Reach, Effectiveness, Adoption, Implementation, Maintenance) [34], its development proceeded in four steps: (1) mapping relevant dimensions from the intervention model, prioritising elements like perceived usefulness of materials, content comprehensibility, and implementation barriers; (2) literature search for validated items, drawing primarily from the validated Learning Effectiveness Survey (LES) [35], which is suitable for assessing material utility and long-term learning outcomes and supplemented by a targeted battery on teaching methods from the Student Assessment of Learning Gains (SALG) [36]; (3) selecting approaches to assess implementation trajectories and contextual influences on effectiveness, and aligning them to intended learning outcomes with a targeted focus on the components that students have worked with (e.g., only Module A, or Module A and C, etc.); (4) designing concise items to evaluate feasibility, didactic strategies, and attitudes without overburdening respondents; (5) evaluating the teaching materials to secure alignment between questionnaire items and possible implementation trajectories.

The final questionnaires were reviewed by the intervention development team to ensure alignment with the intervention objectives, intended learning outcomes, and anticipated implementation pathways. The student PEQ focused on perceived usefulness and comprehensibility of teaching materials, engagement with learning activities, attitudes towards iodine-related health behaviours, and practical barriers to participation. The teacher PEQ focused on implementation feasibility, including resource availability, curriculum compatibility, technical challenges, time constraints, and preparedness to deliver the intervention as intended.

Together with the planned qualitative evaluation interviews, the PEQ was designed to provide a broader understanding of how intervention components were implemented and experienced across study regions.

### 2.8. Planned Statistical Analysis

To evaluate the effects of the intervention over the three timepoints, mixed-effects regression models with random intercepts for region, school, class, and student will be applied to account for the hierarchical and longitudinal structure of the data. Models will assess changes in iodine awareness and iodine-related dietary behaviours while adjusting for age, gender, school type, and socioeconomic status. Interaction analyses and region-specific analyses will further be conducted to explore whether intervention effects differ across population subgroups and study regions.

## 3. Results

### 3.1. Production of Material

**Figure 4:**
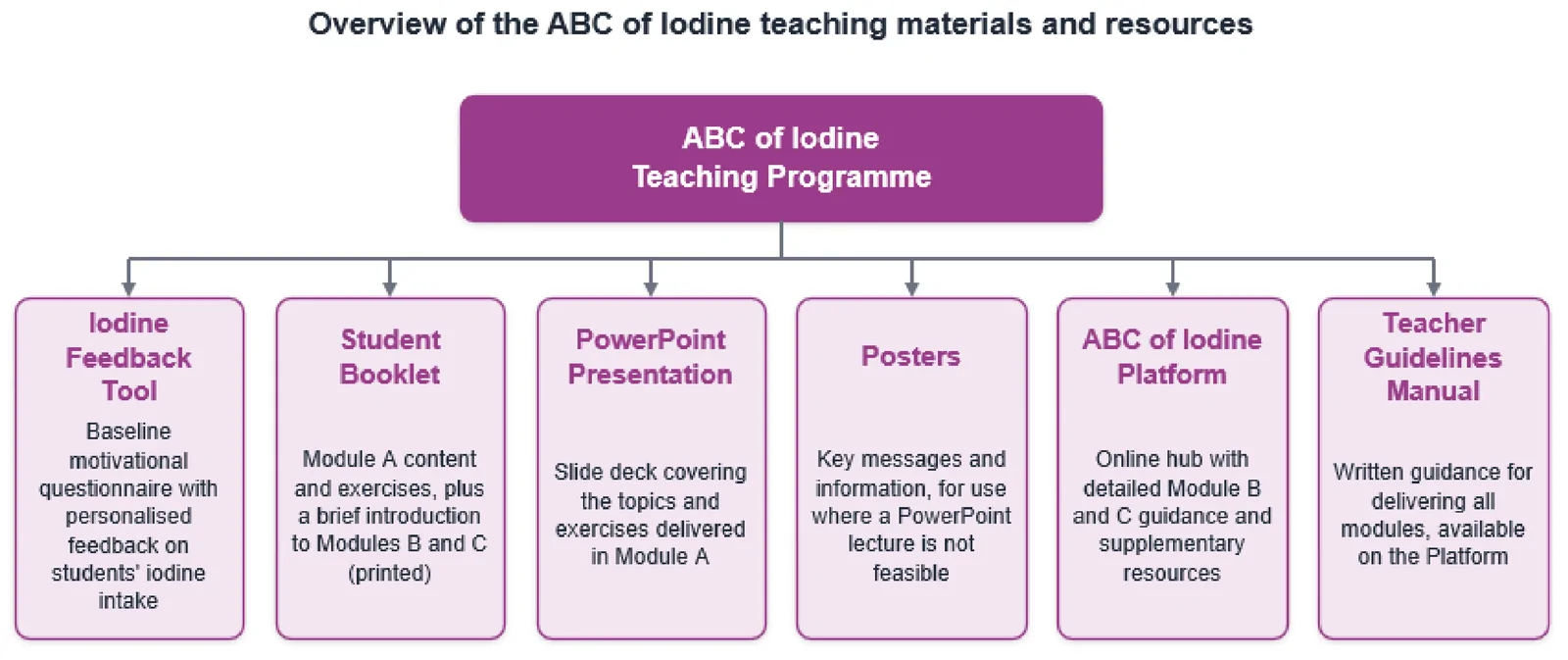
Overview of the materials and resources in *The ABC of Iodine* teaching programme.

**Figure 5:**
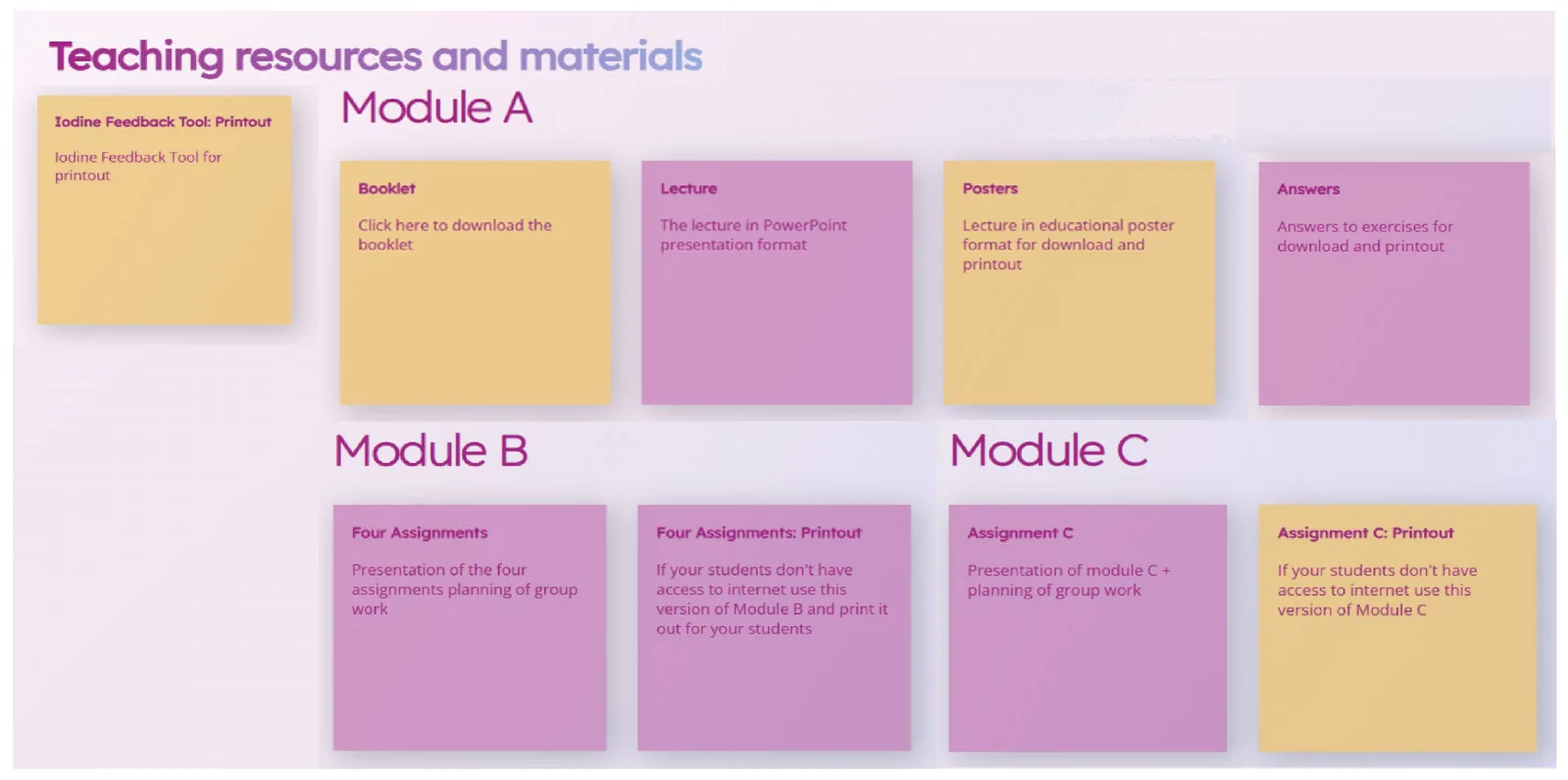
The landing page at *The ABC of Iodine* platform, teachers’ entry providing access to intervention materials and resources for the three teaching modules.

The development process resulted in the following teaching materials and resources: the Iodine Feedback Tool; the booklet containing Module A content and exercises, along with brief introductions to Modules B and C; the PowerPoint presentation outlining the topics and exercises covered in Module A; and four posters with main messages and key information (e.g., for replacement of the PowerPoint presentation); *The ABC of Iodine* platform hosting detailed descriptions and guidelines for Modules B and C, together with Supplementary Materials to support students in completing their assignments (see Figure 4); and a teacher guideline manual that was also available on *The ABC of Iodine* platform (see Figure 5).

### 3.2. Translation and Adaptation of Imagery

**Figure 6:**
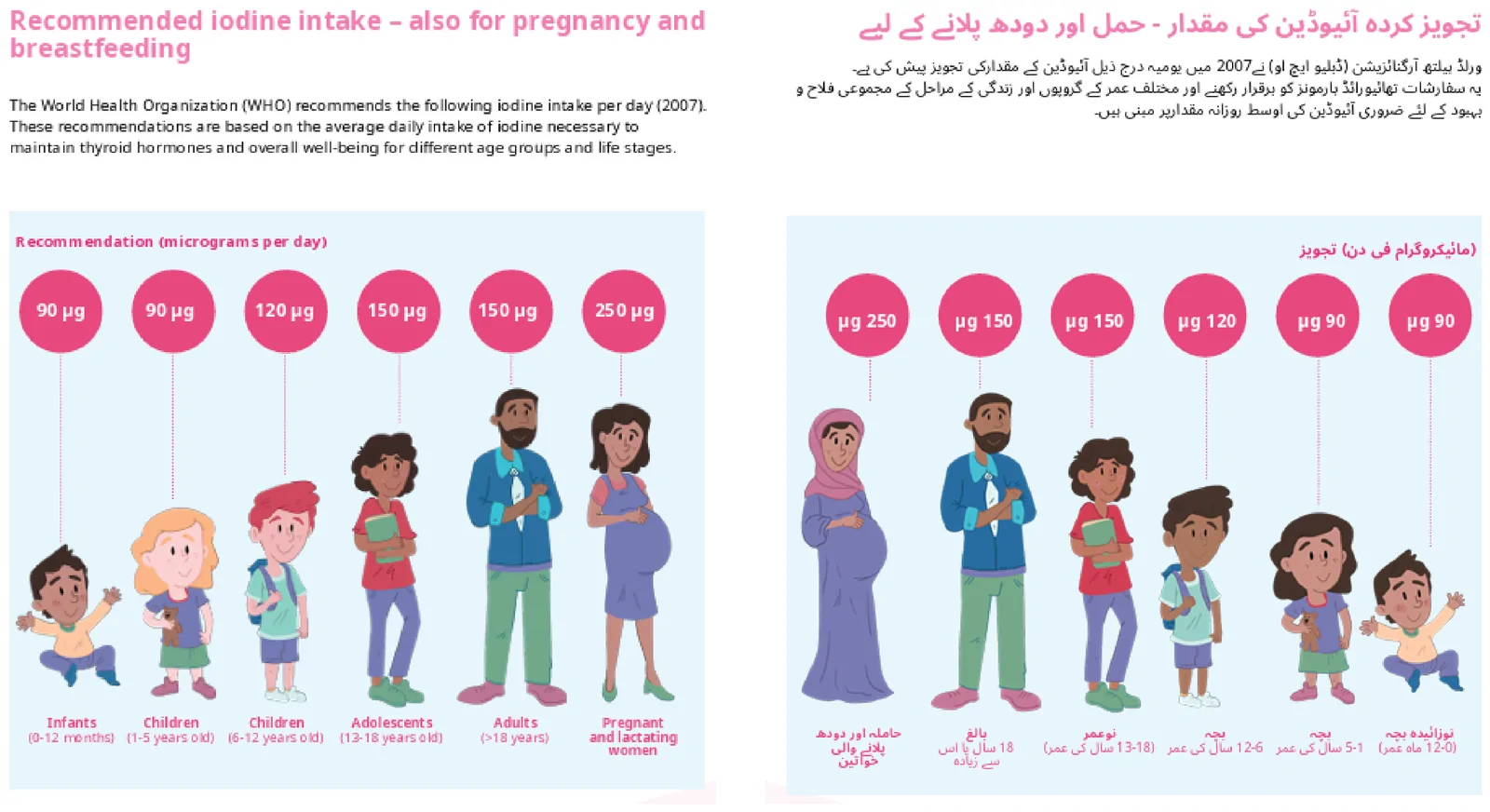
Example of contextual adaptation of educational materials showing the English and Pakistani versions of recommended iodine intake infographic from the student booklet.

**Figure 7:**
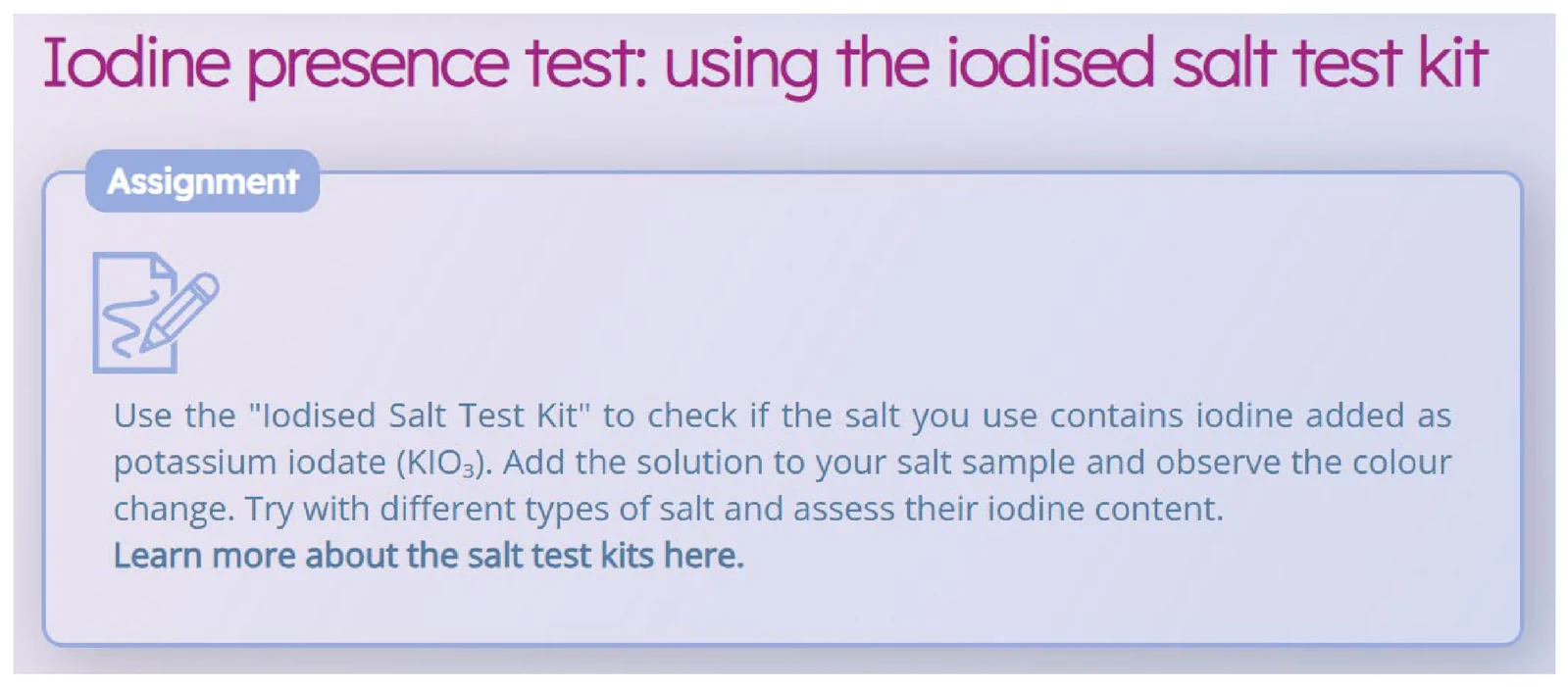
Example of a contextually adapted Module B assignment: instructions for testing the iodine content of household salt samples from the Bengali entry at the *ABC of Iodine* platform.

**Figure 8:**
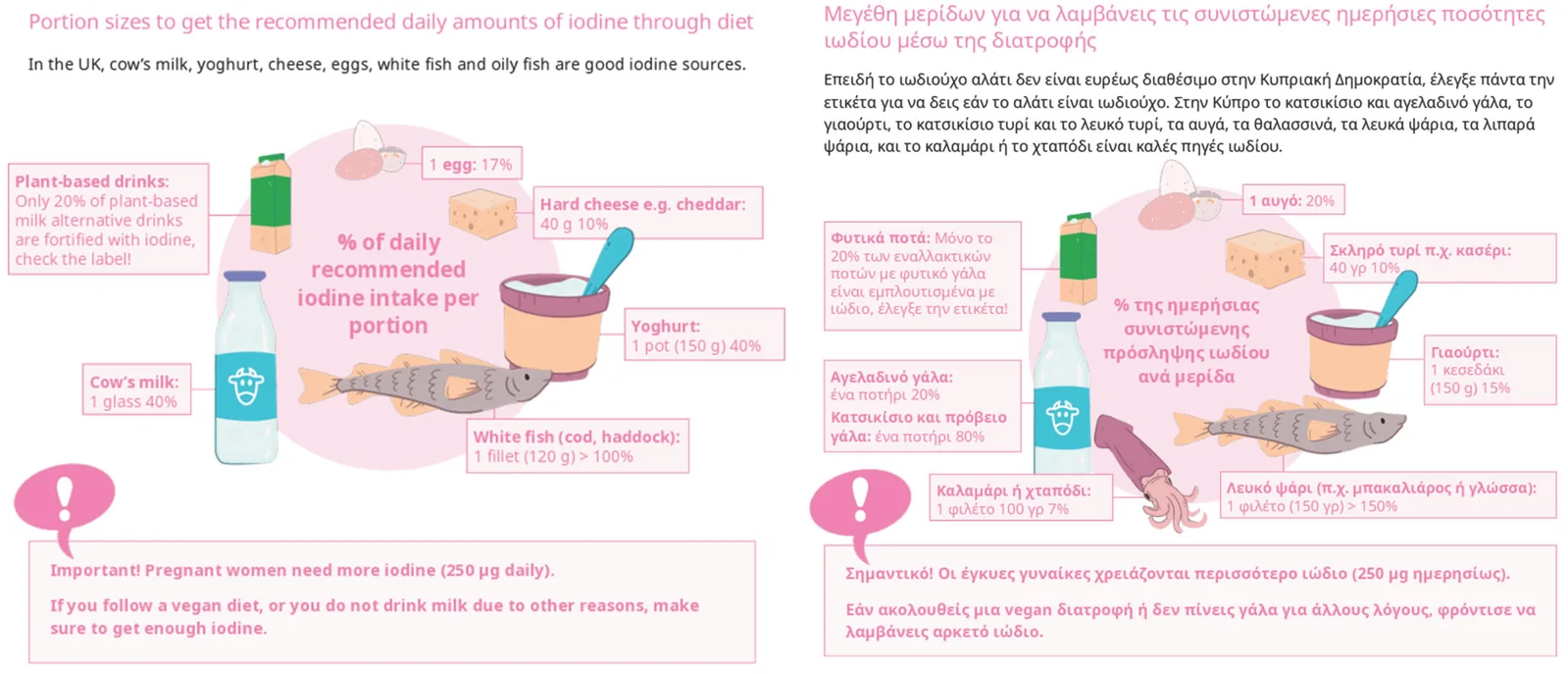
Example of contextual adaptation of booklet content to country-specific dietary sources of iodine in the UK and Cyprus.

All teaching materials and resources were initially developed in English by the design team and subsequently translated into local languages by regional partners (see Figure 6, Figure 7 and Figure 8 for examples), with imagery and visual design adapted to align with cultural contexts and norms.

This iterative process from the development phase ensured optimal tailoring to each study region. In practice, materials varied somewhat across regions, primarily in country-specific guidelines on commonly consumed iodine sources, recommended portion sizes of these sources, and the availability of assignment B5 in Module B (testing iodine content in salt), which was feasible only in Bangladesh.

## 4. Discussion

This intervention’s development illustrated a theoretically grounded, iterative approach to designing complex health education intervention materials. It drew on the Behaviour Change Wheel to target capability, opportunity, and motivation (COM-B) for sustained iodine awareness [22] and was further guided by complex interventions frameworks grounded in implementation research that emphasised contextual adaptation and feasibility assessment [21]. Didactic considerations prioritised motivational triggers, such as perceived relevance and collaborative activities, within a flexible modular structure. This enabled regional tailoring of implementation trajectory and content (e.g., to accommodate curriculum obligations or time constraints) and thereby tested different teaching modalities against predefined intended learning outcomes to assess sustained iodine awareness.

Lessons learned from this process highlighted the value of early stakeholder engagement across project partners and end-users (teachers and implementation staff). Although in practice, their role in funded projects often remains limited to consultation, involving stakeholders as early as possible is critical. Coordinating parallel translations, cultural adaptations, and integrating insights from pre-testing proved demanding, yet it improved the contextual fit and increased the intervention’s potential for improving iodine awareness.

## 5. Conclusions

This intervention demonstrated how theoretically informed and flexible design strategies can be used to support iodine awareness promotion across heterogenous contexts, with iterative adaptations enhancing contextual suitability despite coordination challenges. However, the effectiveness of the teaching programme in improving iodine awareness and related behaviours remains to be established and will be evaluated in the ongoing study. Outcome measures are pending completion of the intervention, and findings are expected to be published at the end of 2026.

## Figures and Tables

**Table 1:** Overview of Intended Learning Outcomes for the *ABC of Iodine* Teaching Programme.

Intended Learning Outcomes
Describe the basic principles of the bodily functions of iodine
Describe iodine’s significance for health and growth
Explain the importance of sufficient iodine intake during pregnancy
List WHO recommendations regarding dietary iodine intake for different age groups
Explain the health consequences of iodine deficiency
Identify good iodine sources in the diet
Apply iodine knowledge in healthy dietary choices

## Data Availability

The data presented in this study are available on request from the corresponding author. The data are not publicly available due to ethical restrictions, as they contain sensitive information from participants under 18 years of age.

## Supplementary Materials

The following supporting information can be downloaded at: https://www.mdpi.com/article/10.3390/nu18172820/s1, Supplementary Material S1: The Iodine Feedback Tool Questionnaire; Supplementary Material S2: Process Evaluation Questionnaire for students; Supplementary Material S3: Process Evaluation Questionnaire for teachers and teaching staff.

## Abbreviations

The following abbreviations are used in this manuscript:

IDD	Iodine Deficiency Disorders
WHO	World Health Organization
BCW	Behaviour Change Wheel
EUthyroid2	European Thyroid Research Programme 2
PEQ	Process Evaluation Questionnaire
