# Supplementary material for: Designing a School-Based, Complex Public Health Intervention to Improve Iodine Awareness in Adolescents in Six Countries
Source: Nutrients. 2026 Aug 28;18(17):2820. doi: 10.3390/nu18172820 (PMC13567452; doi:10.3390/nu18172820)
Supplement: Supplementary file 1 [file nutrients-18-02820-s001.zip › S1_Iodine feedback tool.pdf]

# Iodine Feedback Tool

The following questions are designed to determine whether you are consuming a healthy amount of iodine.

Co-funded by the European Union

UK Research and Innovation

## 1. How many portions of cow's milk and yoghurt do you consume?

One portion= One glass of milk (200 ml) or one pot (125-150 g) with yoghurt.

| | | Points |
|---|---|---|
| Less than three portions/day | ☐ | 0 |
| Three or more portions/day | ☐ | 1 |

## 2. How often do you consume white sea fish?

White fish include fish species such as cod and haddock. It does not include the oily fish species such as salmon, mackerel, and herring.

| | | Points |
|---|---|---|
| Less than two times per week | ☐ | 0 |
| Two or more times per week | ☐ | 1 |

## 3. Do you use dietary supplements containing iodine?

Be aware that the labelling iodine can be given as iodine, iodide, potassium iodide, and sodium iodide. This does not include seaweed or kelp supplements.

| | | Points |
|---|---|---|
| Once a week or never | ☐ | 0 |
| 2-4 times per week | ☐ | 1 |
| 5 times or more per week | ☐ | 2 |
| I am not sure | ☐ | 0 |

## 4. Do you use salt with iodine (labelled as iodised salt) for cooking and baking?

| | | Points |
|---|---|---|
| Never or rarely | ☐ | 0 |
| Several times per week, but not always | ☐ | 1 |
| Yes, I always use salt with iodine | ☐ | 2 |
| I am not sure | ☐ | 0 |

# Iodine Feedback

| | |
|---|---|
| 0-1 points: | Based on your answers, it is a good idea to make more healthy iodine choices. Even small changes may have a great impact on your iodine intake. |
| ≥ 2 points: | Based on your answers, you have healthy iodine habits. It is good to eat a varied diet. |

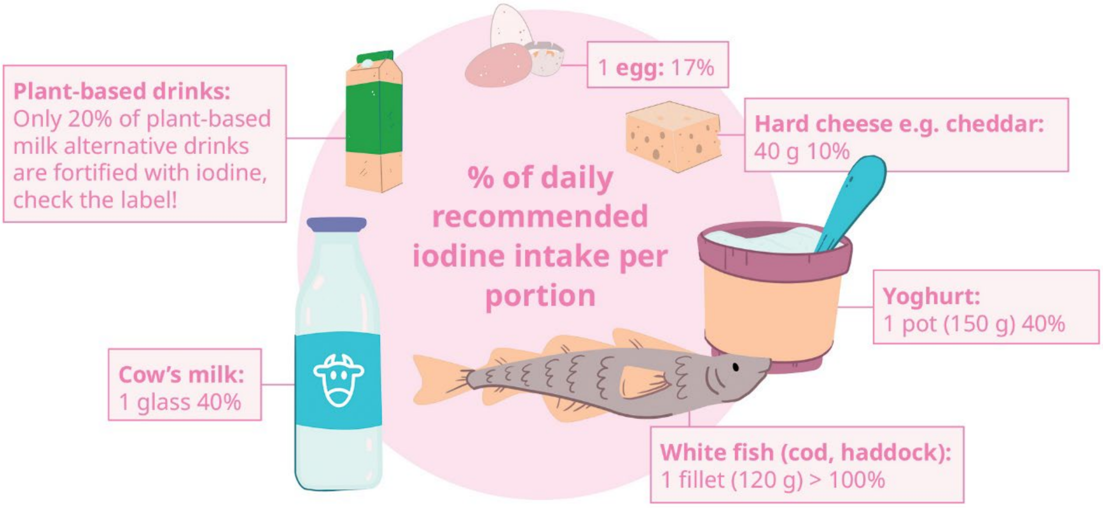


Co-funded by the European Union
UK Research and Innovation
