# Supplementary material for: Designing a School-Based, Complex Public Health Intervention to Improve Iodine Awareness in Adolescents in Six Countries
Source: Nutrients. 2026 Aug 28;18(17):2820. doi: 10.3390/nu18172820 (PMC13567452; doi:10.3390/nu18172820)
Supplement: Supplementary file 1 [file nutrients-18-02820-s001.zip › S2_Process Evaluation Questionnaire_students.pdf]

Questionnaire
Process Evaluation

Dear Students,

We hope that you enjoyed the ABC of Iodine teaching programme. To help us understand how well the teaching materials and approaches worked, we would like to hear your thoughts and experiences. Your feedback is very important to us as it will help us improve the programme for future students.

In this questionnaire, you will be asked about the lesson and different activities that you participated in. We want to know what you liked, what you found helpful, and what could be improved. Please be as honest in your responses as you can. Your answers will remain anonymous – meaning, it will not be possible to tell who gave which answers.

Thank you for your participation!

«barcode»

Co-funded by the European Union
UK Research and Innovation

1. Teaching materials

1.1. Did you attend the lesson on iodine and health?

Yes	☐
Partly	☐
No	☐

1.2. Did you receive your own copy of the booklet: 'The ABC of Iodine'?

Yes	☐
Yes, but I don't have it anymore	☐
No	☐

1.3 Did you do the exercises for module A in the booklet (pages 17-19)

Yes	☐
Partly	☐
No	☐

1.4. Did you do one or more of the assignments in module B? Please tick all the assignments you have completed.

B1: Iodine awareness quest: crafting a community questionnaire	☐
B2: Iodine detective: exploring family food records	☐
B3: Tracing iodine: mapping global fortification programmes	☐
B4: Iodine investigation: exploring local food sources	☐

If you selected one or more of the answers in 1.4, please answer 1.4.A.
If you selected none of the answers, please continue with question 1.5.

1.4.A Did you use one or more of the online resources provided at the Learning Platform for the assignment(s) in Module B?

	1	2	3	4	5	
**To a large extent**	O	O	O	O	O	**Not at all**

«barcode»

1.4.B Did you have the chance to work with one or more classmates in completing the assignment(s) in Module B?

Yes	☐
Partly	☐
No	☐

If you selected **Yes or Partly** in 1.4 B, please answer 1.4.B.1
If you selected **No**, please continue with question 1.5.

1.4 B1 The joint learning activities were fun and inspiring.

Completely agree	☐
Agree	☐
Neither agree nor disagree	☐
Disagree	☐
Completely disagree	☐

1.5 Did you develop and design a poster for the imaginary iodine campaign in module C?

Yes	☐
Partly	☐
No	☐

If you selected **Yes** or **Partly** in 1.5, please answer 1.5.A.
If you selected **No**, please continue with question 2.1

1.5.A Did you have the chance to work with one or more classmates in developing and designing the poster?

Yes	☐
Partly	☐
No	☐

If you selected **Yes** or **Partly** in 1.5.A, please answer 1.5.A.1.

If you selected **No**, please continue with question 2.1

1.5.A.1 The joint learning activities were fun and inspiring.

Completely agree	☐
Agree	☐
Neither agree nor disagree	☐
Disagree	☐
Completely disagree	☐

«barcode»

2. Learning

2.1 Did you learn something new about iodine that you did not already know?

Yes	☐
No	☐

2.2 The teacher was well prepared to lead in-class activities.

Completely agree	☐
Agree	☐
Neither agree nor disagree	☐
Disagree	☐
Completely disagree	☐

2.3 Please answer the following questions about the statement(s) given:

	A great deal	A lot	A moderate amount	A little	Not at all
How much did the lesson in module A contribute to your learning?	☐	☐	☐	☐	☐
How much did the booklet contribute to your learning?	☐	☐	☐	☐	☐
How much did the exercises in module A contribute to your learning?	☐	☐	☐	☐	☐

«barcode»

If you did one or more of the assignments in module B, please answer 2.4

If you did not do any assignments in module B, please continue with question 2.5.

2.4 Please answer the following questions about the statement(s) given:

	A great deal	A lot	A moderate amount	A little	Not at all
How much did the assignments in module B contribute to your learning?	☐	☐	☐	☐	☐
How much did the online resources for module B provided at the Learning Platform contribute to your learning?	☐	☐	☐	☐	☐

If you did develop and design a poster, please answer 2.5

If you did not develop and design a poster, please continue with question 2.6.

2.5 Please answer the following questions about the statement(s) given:

	A great deal	A lot	A moderate amount	A little	Not at all
How much did the work with the poster for the imaginary iodine campaign in module C contribute to your learning?	☐	☐	☐	☐	☐
How much did the online resources for module C provided at the Learning Platform contribute to your learning?	☐	☐	☐	☐	☐

2.6 Do you think the lesson, exercises, and assignments will influence how much attention you pay iodine in your diet in the future?

Definitely	☐
Probably	☐
Maybe	☐
Probably not	☐
Definitely not	☐

«barcode»

3. Usability

3.1 Please answer the following with your level of agreement to the given statement(s):

The booklet …	Completely agree	Agree	Neither agree nor disagree	Disagree	Completely disagree
…is easy to understand.	☐	☐	☐	☐	☐
…helped me to understand iodine deficiency.	☐	☐	☐	☐	☐
…has given me confidence to make sure that I have a healthy iodine intake.	☐	☐	☐	☐	☐
…design was appealing to me (e. g. illustrations, colours structure).	☐	☐	☐	☐	☐

3.2 Please answer the following with your level of agreement to the given statement(s):

The lesson …	Completely agree	Agree	Neither agree nor disagree	Disagree	Completely disagree
…was easy to understand.	☐	☐	☐	☐	☐
…helped me to understand iodine deficiency.	☐	☐	☐	☐	☐
…has given me confidence to make sure that I have a healthy iodine intake.	☐	☐	☐	☐	☐
…design was appealing to me (e. g. illustrations, colours structure).	☐	☐	☐	☐	☐

«barcode»

3.3 Please answer the following with your level of agreement to the given statement(s):

	Completely agree	Agree	Neither agree nor disagree	Disagree	Completely disagree
The instructions for the assignments were clear and easy to understand.	☐	☐	☐	☐	☐
The assignments were easy for me to do.	☐	☐	☐	☐	☐
The online resources helped with the assignments (module B and/or C).	☐	☐	☐	☐	☐
The structure of the Learning Platform was clear and understandable, e.g. it was easy to find what I was looking for.	☐	☐	☐	☐	☐

3.4. Did you encounter any technical problems, e.g. access to the Learning Platform, the Iodine Feedback Tool or other? (Multiple choices possible)

No, I had no technical issues.	☐
Yes, an unstable internet connection caused problems.	☐
Yes, I could not get access to the Learning Platform.	☐
Yes, I could not get access to the Iodine Feedback Tool.	☐
Other	☐

Other (specify in text)

«barcode»

3.5 Do you have suggestions for improvements of the lesson, teaching materials, preparation resources, or other?

«barcode»

QUEEN'S UNIVERSITY BELFAST

UNIVERSITY OF SURREY

EUthyroid2

This is the end of the questionnaire.

THANK YOU for filling it out!

«barcode»

Co-funded by the European Union

UK Research and Innovation
