# Supplementary material for: Designing a School-Based, Complex Public Health Intervention to Improve Iodine Awareness in Adolescents in Six Countries
Source: Nutrients. 2026 Aug 28;18(17):2820. doi: 10.3390/nu18172820 (PMC13567452; doi:10.3390/nu18172820)
Supplement: Supplementary file 1 [file nutrients-18-02820-s001.zip › S3_Process Evaluation Questionnaire_teachers and teaching staff.pdf]

Questionnaire
Process Evaluation

Dear Teacher,

We hope that you enjoyed the ABC of Iodine teaching programme. To help us understand how well the teaching materials and approaches worked, we would like to hear your thoughts and experiences. Your feedback is very important to us as it will help us improve the programme for future teachers.

In this questionnaire, you will be asked about the lesson and different activities that you planned and taught. We want to know what you liked, what you found helpful, and what could be improved. Please be as honest in your responses as you can. Your answers will remain anonymous.

Thank you for your participation!

«barcode»

Co-funded by the European Union
UK Research and Innovation

1. Modules and Materials

1.1. Did you use module A (lesson, exercises, and tasks) in your teaching?

Yes	☐
Partly	☐
No	☐

If you selected **Yes** or **Partly** in 1.1, please answer 1.1.A.

If you selected **No**, please continue with question 1.2

1.1.A How much time did you spend teaching Module A (holding the lesson, exercises, and tasks)

Less than 30 minutes	☐
30-59 minutes	☐
60-89 minutes	☐
90-120 minutes	☐
More than 120 minutes	☐

1.2 Did you use Module B (assignments) in your teaching?

Yes	☐
Partly	☐
No	☐

If you selected **Yes** or **Partly** in 1.2, please answer 1.2.A.

If you selected **No**, please continue with question 1.3

1.2.A Which assignment(s) did you incorporate in your teaching?

Assignment B1: Iodine awareness quest: crafting a community questionnaire	☐
Assignment B2: Iodine detective: exploring family food records	☐
Assignment B3: Tracing iodine: mapping global fortification programmes	☐
Assignment B4: Iodine investigation: exploring local food sources	☐

«barcode»

1.3 Did you use Module C (crafting posters) in your teaching?	
Yes	☐
No	☐

1.4 Which parts of the teaching materials did you use in your teaching? (Select all that apply)	
The booklet	☐
The lesson in PowerPoint format	☐
The lesson in poster format	☐
The Iodine Feedback Tool	☐

1.5 Did you adapt the materials in Module A, B and/or C to better suit your teaching style or students' needs?	
Yes	☐
No	☐

If you selected **Yes** in 1.5, please answer 1.5.A.
If you selected **No**, please continue with question 2.1

1.5.A Why and how did you adapt the materials?

«barcode»

2. Preparation

2.1. In which subject did you implement the ABC of Iodine teaching programme? Choose all that apply.

Biology	☐
Chemistry	☐
Health & Social Care	☐
Mathematics	☐
Home Economics	☐
Art and Design	☐
Other, please specify	☐

Other:

2.2 How did the ABC of Iodine teaching programme fit into the curriculum of the subject(s)?

Very well	☐
Well	☐
Neutral	☐
Poorly	☐
Not at all	☐

2.3 How much time did you spend preparing your teaching?

Less than 30 minutes	☐
30-90 minutes	☐
More than 90 minutes	☐

«barcode»

2.4 Please answer the following with your level of agreement to the given statement: I had the time I needed to prepare my teaching.

Completely agree	☐
Agree	☐
Neither agree nor disagree	☐
Disagree	☐
Completely disagree	☐

2.5 I used the following in my preparation for the class:

One or more instruction videos on the Learning Platform	☐
Teachers' guidelines	☐
The booklet	☐
Slides and notes in the PowerPoint presentation	☐
Information on iodine and health I searched for online	☐
Other: (specify in text)	☐

Other:

2.6 Please answer the following with your level of agreement to the given statement(s):

	Completely agree	Agree	Neither agree nor disagree	Disagree	Completely disagree
I had access to the materials, guidelines, and other resources that I needed for my planning.	☐	☐	☐	☐	☐
The structure of the Learning Platform is clear and understandable, e.g. it is easy to find what I am looking for.	☐	☐	☐	☐	☐
I felt well prepared for my teaching.	☐	☐	☐	☐	☐

«barcode»

3. Usability

3.1 Please answer the following with your level of agreement to the given statement(s):

	Completely agree	Agree	Neither agree nor disagree	Disagree	Completely disagree
The teachers' guidelines are comprehensible.	☐	☐	☐	☐	☐
I did not read the teachers' guidelines.	☐				
The lesson's contents and exercises are comprehensible.	☐	☐	☐	☐	☐
I did not hold the lesson.	☐				
The booklet's contents are comprehensible.	☐	☐	☐	☐	☐
I did not read the booklet.	☐				
The instructions for the assignments are clear and easy to understand.	☐	☐	☐	☐	☐
I did not read the instructions for the assignments.	☐				
The assignments are easy to do for the majority of students.	☐	☐	☐	☐	☐
I did not use the assignments in my teaching.	☐				

«barcode»

3.2 Did you encounter any technical issues? (Multiple answering options possible)	
No, I had no technical issues.	☐
Yes, an unstable internet connection caused problems.	☐
Yes, I could not get access to the Learning Platform.	☐
Yes, I could not download and print materials from the Learning Platform.	☐
Yes, I could not get access to the Iodine Feedback Tool.	☐
Other	☐

Other, please explain here

«barcode»

4. Context

4.1 Please answer the following with your level of agreement to the given statement(s):

	Completely agree	Agree	Neither agree nor disagree	Disagree	Completely disagree
The school leadership actively supported that I implemented the ABC of Iodine teaching programme in my teaching.	☐	☐	☐	☐	☐
I felt confident in my ability to integrate the ABC of Iodine teaching programme into my teaching.	☐	☐	☐	☐	☐
I had sufficient time available to prepare and implement the ABC of Iodine teaching programme.	☐	☐	☐	☐	☐
Additional resources (e.g., training, support) would have helped me use the materials more effectively.	☐	☐	☐	☐	☐

4.2 Please specify what kind of resources you would have benefitted from:

«barcode»

5. System Impact

5.1. Have you discussed the topic of iodine with colleagues, or others?

No	☐
Yes, once	☐
Yes, several times	☐

5.2 Please answer the following questions about the statement(s) given:

	Very likely	Likely	Neutral	Unlikely	Very unlikely
How likely is it that you will teach other classes the topic of iodine **after** the ABC of Iodine project has ended?	☐	☐	☐	☐	☐
How likely is it that you will use (part of) the teaching materials to teach other classes **after** the ABC of Iodine project has ended?	☐	☐	☐	☐	☐

5.3 To what extent do you believe the project can be scaled to other schools and educational institutions teaching adolescents aged 13-17 years?

	**1**	**2**	**3**	**4**	**5**	
**To a large extent**	O	O	O	O	O	**Not at all**

«barcode»

5.4 How would you rate the overall content of the ABC of Iodine teaching programme?	
Excellent	☐
Good	☐
Average	☐
Below average	☐
Poor	☐

5.5 How feasible did you find the implementation of the ABC of Iodine teaching programme in your school?	
Very infeasible	☐
Somewhat infeasible	☐
Neutral	☐
Somewhat feasible	☐
Very feasible	☐

5.6 In your opinion, how effective is the ABC of Iodine teaching programme in helping students acquire knowledge about iodine?	
Very ineffective	☐
Ineffective	☐
Neutral	☐
Effective	☐
Very effective	☐

«barcode»

5.7 Please answer the following with your level of agreement to the given statement:

I personally found implementing the ABC of Iodine teaching programme enjoyable and engaging.

Completely agree	☐
Agree	☐
Neither agree nor disagree	☐
Disagree	☐
Completely disagree	☐

5.8 Do you have suggestions for improvements of the lesson, teaching materials, preparation resources, or other?

«barcode»

EUthyroid2

This is the end of the questionnaire.

THANK YOU for filling it out!

«barcode»

Co-funded by the European Union

UK Research and Innovation
